# Supplementary material for: Fly Ash from Lignite Combustion as a Filler for Rubber Mixes. Part I: Physical Valorization of Fly Ash
Source: Materials (Basel). 2022 Jul 13;15(14):4869. doi: 10.3390/ma15144869 (PMC9324915; doi:10.3390/ma15144869)
Supplement: Supplementary file 1 [file materials-15-04869-s001.zip › materials-1757626-supplementary.pdf]

28th of June, 2022

A)

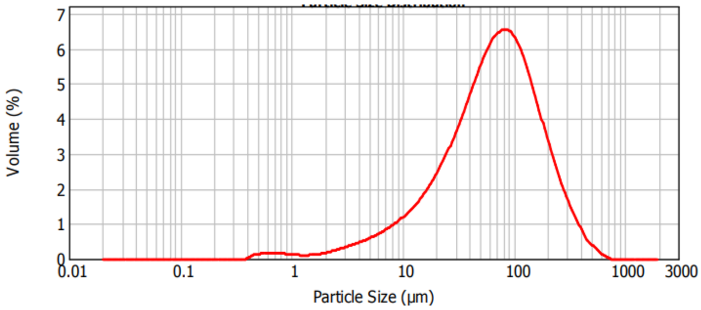

B)

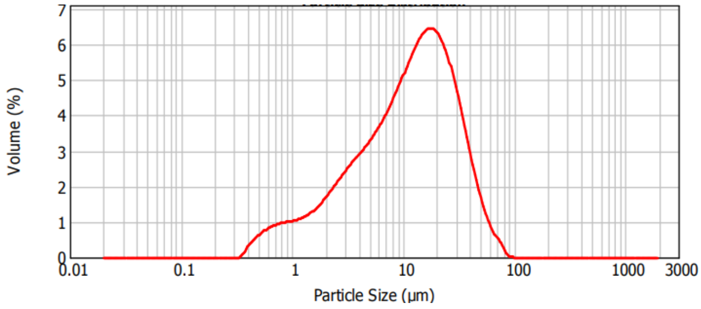

Figure S1. Particle size distribution analysis for fly ash sample FA-B 2018: A) before grinding, B) after 4 hrs grinding.

A)

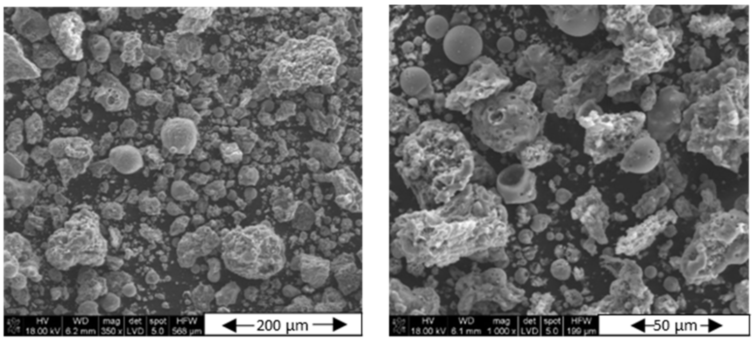

B)

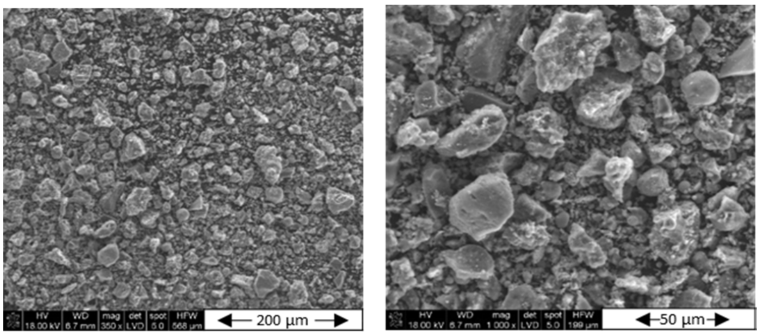

Figure S2. SEM morphology of the fly ash sample FA-B 2018: A) before grinding, B) after 4 hrs grinding.

Table S1. Qualitative and quantitative "oxide" analysis of fly ash sample FA-B 2018.

No.	Component	Content [wt. %]
1	SiO_2	37.7
2	Al_2O_3	26.4
3	CaO	20.9
4	Fe_2O_3	7.38
5	TiO_2	0.89
6	SO_3	2.74
7	MgO	1.01
8	K_2O	0.11
9	P_2O_5	0.44
10	SrO	0.06
11	BaO	0.05
12	Mn_3O_4	0.05
13	Na_2O	0.04
SUM		99.03
Loss on Ignition [wt. %]		1.26

Table S2. Phase composition for fly ash sample FA-B 2018.

**No.**	**Component**	**Content [wt. %]**
1	Low silicon dioxide (SiO_2)	14.3
2	Akermanite ($Ca_2Mg[Si_2O_7]$)-<br>Gehlenite ($Ca_2Al[(Si,Al)_2O_7]$)	41.8
3	Andesine<br>Sodium aluminosilicte<br>($NaAlSi_3O_8$) and<br>Calcium aluminosilicate<br>($CaAl_2Si_2O_8$)	29.6
4	Potassium aluminosilcate<br>($AlKO_6Si_2$)	6.0
5	Mayenite ($Ca_{12}Al_{14}O_3$)	6.3
6	Leucite ($K[AlSi_2O_6]$)	1.0
7	Zeolite MCM-70	1.1

Table S3. SEM-EDS analysis of the chemical composition of the fly ash FFA-B 2018 fractions in terms of the content of oxides and carbon.

Grain size/fraction	**Σ oxides [wt. %]**	**Carbon [wt. %]**
FA-63	85.6	14.4
FA-125	72.7	27.3
FA-250	62.9	37.1
FA>0.250	49.1	50.9

Table S4. "Oxide" analysis of the fly ash FFA-B 2018 fractions.

Fraction/ component	**FA-63 [wt. %]**	**FA-125 [wt. %]**	**FA-250 [wt. %]**	**FA>0.250 [wt.%]**
SiO_2	20.618	25.223	25.471	18.207
CaO	27.941	17.489	11.772	9.245
Al_2O_3	19.160	18.911	16.865	11.620
Fe_2O_3	8.897	5.931	4.753	4.168
SO_3	6.076	2.462	1.534	3.213
TiO_2	0.941	1.192	1.246	1.239
MgO	0.914	0.065	0.459	0.326

Table S5. Phase composition of various fractions of the fly ash FFA-B 2018 sample.

Fraction/ Component	**FA-63 [wt.%]**	**FA-125 [wt.%]**	**FA-250 [wt.%]**	**FA>0.250 [wt.%]**
Quartz (SiO_2)	10.0	23.8	39.2	46.9
Akermanite/ Gehlenite $Ca_2Mg(Si2O_7)$ $Ca_2Al[(Si_2Al)_2O_7]$	30.9	13.1	9.1	4.1
Albite $NaAlSi_3O_8$	20.0	24.0	19.2	11.7
Limestone CaO	6.6	1.3	-	-
Calcite $CaCO_3$	2.2	1.3	1.2	-
Hematite α-Fe_2O_3	5.0	1.6	1.3	-
Anhydrite $CaSO_4$	10.0	2.2	2.1	1.8
Brownmillerite $Ca_2(Al_2Fe)_2O_5$	6.0	1.7	2.0	-
Mullite $3Al2O_3{\cdot}2SiO2$	9.4	30.9	25.9	35.5

Table S6. Specific surface area analysis of fly ash FA-B 2018 sample.

Sample	**CTAB [m^2/g]**	**BET [m^2/g]**	**BET [m^2/g] after grinding**
FA-B	4.0±0.1	11.1±0.1	11.3±0.1

Table S7. Results of the BET surface area for fly ash FFA-B 2018 sample divided into fractions.

Fraction	**BET [m^2/g]**
FA-63	5.3
FA-125	10.5
FA-250	17.6
FA>0.250	45.1

Table S8. Surface energy of fly ash FA-B 2018 sample.

Surface energy [mJ/m^2]	
Dispersive component	22.6
Polar component	11.7
Total value	34.3
